# Estrogen and bacterial infection

**DOI:** 10.3389/fimmu.2025.1556683

**Published:** 2025-04-29

**Authors:** Longyan Hong, Hao Liang, Wenqing Man, Yinghui Zhao, Pengbo Guo

**Affiliations:** ^1^ The First Affiliated Hospital of Shandong First Medical University, Shandong Provincial Qianfoshan Hospital, Jinan, China; ^2^ Department of Pathogen Biology, School of Clinical and Basic Medicine, Shandong First Medical University and Shandong Academy of Medical Sciences, Jinan, China; ^3^ Department of Health Inspection and Quarantine, School of Public Health, Cheeloo College of Medicine, Shandong University, Jinan, China; ^4^ School of Stomatology, Shandong First Medical University and Shandong Academy of Medical Sciences, Jinan, China

**Keywords:** estrogen, bacterial infection, sex difference, immune response, molecular mechanism

## Abstract

Gender differences exist in the susceptibility, incidence, progression, and prognosis of bacterial infections in males and females, influenced by various factors including lifestyle and habits. Multiple reports have indicated that estrogen plays a crucial immunomodulatory role in many pathogenic microbial infections, highlighting a complex relationship between estrogen, its receptors, and bacterial infections. Estrogen and its receptors regulate host immune responses, affecting the host’s ability to clear bacteria and thus influencing the likelihood and difficulty of infection eradication. Variations in estrogen levels may lead to differences in the occurrence and progression of bacterial infections, with estrogen playing varied roles in diseases caused by the same bacterial pathogens. The interaction between estrogen and bacterial infections represents a complex and crucial aspect of human physiology and clinical medicine. Understanding this interaction is essential for advancing infection prevention and treatment strategies. This article reviews the correlation and mechanisms between estrogen and bacterial infections, emphasizing the importance of further research in this field.

## 1 Introduction

Bacterial infections pose a common yet significant health challenge, continuously impacting global human health (1). Gender differences play a crucial role in the incidence, severity, and prognosis of bacterial infections, thus attracting widespread attention to the role of gender factors in infectious pathology (2). Over the past few decades, researchers have identified numerous physiological and immunological differences related to gender (3, 4). However, in recent years, there has been a growing recognition of the role of estrogen in modulating bacterial infections (5). Estrogen not only plays a pivotal role in the female reproductive system but also exerts diverse physiological functions in other organs and systems (6–8). Increasing evidence has suggested that estrogen and its receptors not only influence the host’s susceptibility to bacterial infection and clearance capabilities but also directly or indirectly impact bacterial growth, survival, and virulence (4, 9). Understanding the mechanisms of interaction between estrogen and bacterial infections is crucial for developing more effective prevention and treatment strategies. Therefore, this paper aims to review the latest research progress on the role of estrogen in modulating bacterial infections, with the hope of providing new insights and directions for future studies.

## 2 Estrogen

Estrogen, a growth-inducing sex hormone, is expressed in both males and females and exerts its effects through binding to estrogen receptors (ER) α, β (ERα/β), as well as G protein-coupled estrogen receptor (GPER) (10, 11). The interaction between estrogen and its receptors is integral to immune regulation and disease development. They influence the host’s susceptibility to various pathogens, regulate inflammatory responses, influence the differentiation and function of immune cells, and contribute to the initiation and progression of autoimmune diseases (12–15). In bacterial infections, estrogen and its receptors could impact the recognition and clearance of bacteria by immune cells, as well as influence the development and progression of such infections by affecting inflammatory cytokines and signaling pathways. (Effects of Estrogen on Bacteria as Seen in **Supplementary Materials Tables 1**, **2**).

## 3 Estrogen and bacteria

### 3.1 Chlamydia trachomatis

*C. trachomatis*, a Gram-negative bacterium, primarily parasitizes within eukaryotic cells (16). This bacterium is the main pathogen causing trachoma and is a common pathogen of sexually transmitted diseases globally. Infections of *C. trachomatis* in the urogenital tract could lead to various diseases, including urethritis, cervicitis, and pelvic inflammatory disease (17). Furthermore, it can result in severe complications such as tubal infertility and ectopic pregnancy, posing serious health issues to patients (18). Estrogen plays a crucial role in the female reproductive system, and variations in estrogen levels may affect the structure and immune function of the reproductive tract, thereby increasing the risk of *C. trachomatis* infection (19). Studies have shown an association between estrogen and its related receptors with an increased incidence of *C. trachomatis* infection (20–23).

**Figure 1:**
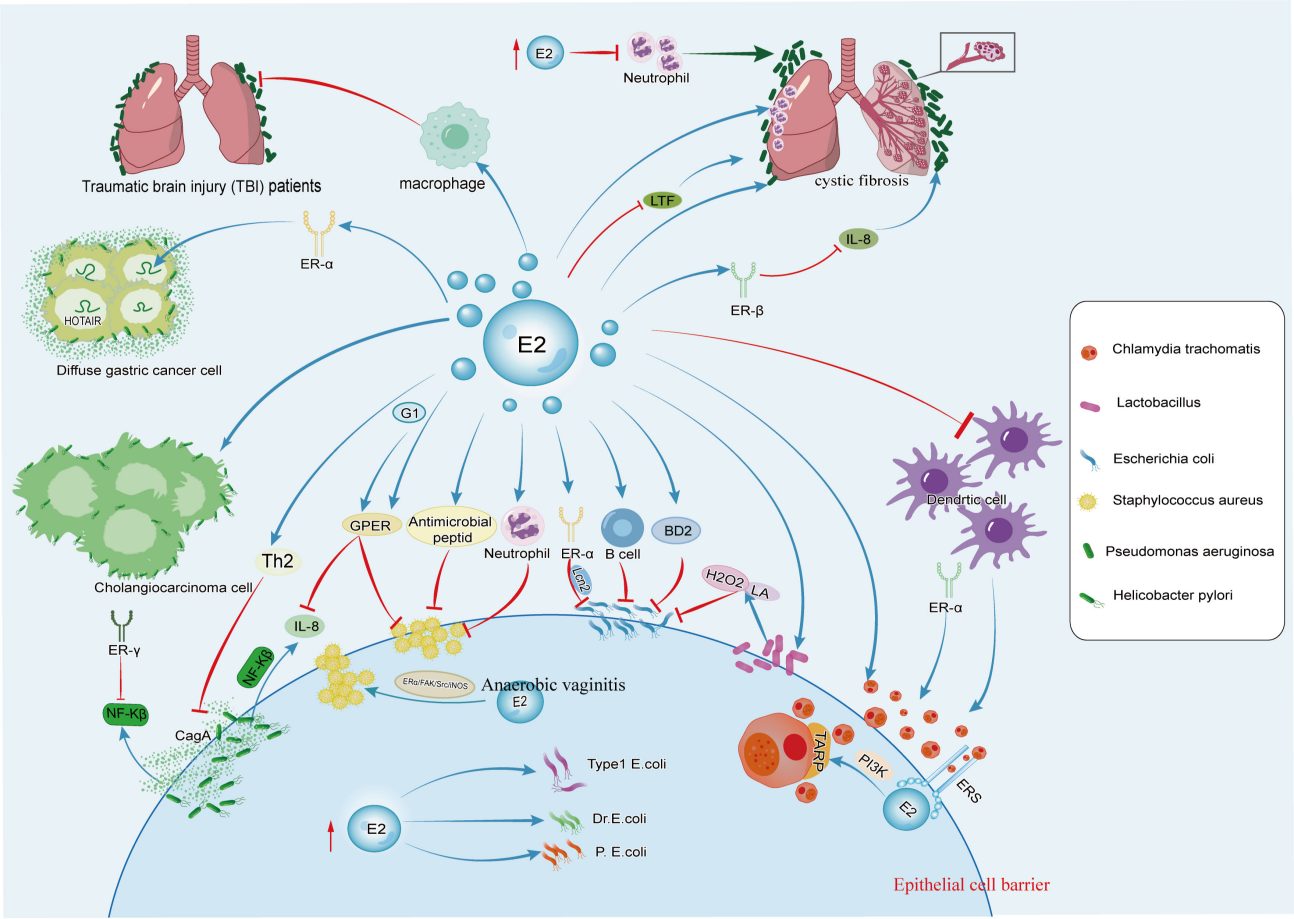
The figure illustrates the effects of estrogen on *C. trachomatis*, *E. coli*, *Lactobacillus*, *S. aureus*, *P. aeruginosa*, and *H. pylori*. Estrogen promotes the growth of *C. trachomatis* and *Lactobacillus* and can also synergize with *P. aeruginosa* to accelerate the occurrence and development of pulmonary cystic fibrosis. It has dual effects on *S. aureus*, *H. pylori*, and *E. coli*.

Membrane estrogen receptors (mERs) facilitate the entry of *C. trachomatis* into host cells, and mER signaling promotes the development of inclusions in *C. trachomatis* infection. Hormones that enhance the *C. trachomatis* infection also involve stromal signals and direct stimulation of uterine epithelial cells by estrogen (21). Activation of mERs leads to the activation of the phosphoinositide 3-kinase (PI3K) signaling pathway, which is involved in cell proliferation. The *C. trachomatis* III effector molecule TARP interacts with PI3K to promote infection. Estradiol also affects the expression of TLR4 and downstream signaling molecules (IRAK4 and Nuclear factor κB), reducing the gene expression of Th1-related cytokines IL-12, IL-6, TNFα, and IFNγ, resulting in incorrect recognition of *C. trachomatis* by dendritic cells and thus increasing susceptibility to *C. trachomatis* infection (22) (**Figure 1**).

After *C. trachomatis* infection, inhibitors of estrogen receptor signaling reduces epithelial ER signaling exposure, affecting the MAPK signaling pathway, including ERK phosphorylation, regulation of downstream effector cPLA2, and the PI3K/AKT signaling pathway and calcium mobilization, all crucial for *C. trachomatis* inclusion development (19, 21).

During *C. trachomatis* infection, the lack of ERα alters the shedding of *C. trachomatis* in the vagina. Compared to WT animals expressing both ERs or only ERα, the progression of *C. trachomatis* infection is faster, and the clearance from the reproductive tract is more rapid in mice with only ERβ (ERαKO) (20) (**Figure 1**). ERα regulates T cell responses to *C. trachomatis* infection in mice. In ERαKO mice infected with *C. trachomatis*, the expression of T cells, FOXP3, and IFNϵ significantly increase. The defect of ERα expression increases stimulation of CD4^+^ T cells in mice’s macrophages. FOXP3, a transcription factor, is a critical marker of regulatory T cells (Tregs), indicating that Tregs are also involved in the clearance of *C. trachomatis* in condition of ERα deficiency (20).

*C. trachomatis* infection is also considered as a major cause of recurrent spontaneous abortion (24). Infection of the trophoblast layer by *C. trachomatis* impairs cellular cholesterol biosynthesis, depleting substrates for estrogen and progesterone synthesis, affecting functions such as implantation and placental formation in the trophoblast layer, thereby influencing pregnancy outcomes (25, 26). Studies have found that decreased progesterone levels lead to increased expression of pro-inflammatory cytokines in recurrent spontaneous abortion associated with infection, resulting in miscarriage by disrupting maternal tolerance of the embryo/fetus (27). Higher susceptibility has been observed during the proliferative phase of the menstrual cycle when estrogen levels are elevated, while progesterone enhances the innate immune response to *C. trachomatis* infection, thereby reducing susceptibility to *C. trachomatis* infection (28, 29).

### 3.2 Lactobacillus

*Lactobacillus*, representative microorganisms of normal vaginal flora, are characterized by being Gram-stain positive, microaerophilic, acid-resistant, non-spore-forming, and capable of producing lactic acid. In the vaginal microbiota of healthy women, *Lactobacillus* dominates, accounting for over 90% of the total vaginal flora (30, 31). Maintaining an acidic microenvironment of the vagina inhibited the growth of pathogens, including *C. trachomatis*, *Neisseria gonorrhoeae* (*N. gonorrhoeae*), and *Escherichia coli* (*E. coli*) *(* 32).

During puberty, the increase in circulating estrogen promotes the proliferation of *lactobacilli*, leading to the production of lactic acid and hydrogen peroxide, which inhibited the growth of pathogens (33). Previous studies have shown that some postmenopausal patients who take estradiol could maintain vaginal pH around 4.5 and normal serum estradiol levels (34). The hormonal imbalance during the female reproductive cycle leads to bacterial vaginosis (BV), with the imbalance of vaginal microbiota playing a role.

The incidence of urinary tract infections begin to rise during menopause, and recurrent urinary tract infections are considered a feature of genitourinary syndrome of menopause, characterized by thinning of the vaginal epithelium, various symptoms associated with vaginal atrophy, and a relative loss of *lactobacilli* in the vaginal microbiota (35). The loss of estrogen during menopause lead to changes in the vaginal microbiota of women, with a decrease in the relative abundance of *lactobacilli* (36). Studies have shown that colonization of *E. coli* is more frequent in women who have not received estrogen replacement therapy, and it is negatively correlated with the presence of *lactobacilli* (37, 38) (**Figure 1**). Estrogen stimulates the proliferation of *lactobacilli* in the vaginal epithelium, reduces the pH value, prevents excessive colonization of intestinal bacteria in the vagina, and thus prevents urinary tract infection (39).

The use of local estrogen preparations to treat menopause-related estrogen deficiency can reduce the incidence of recurrent urinary tract infection in most women and restore *lactobacilli* in the vagina (35). Estrogen also lowers the pH value by stimulating the vaginal mucosa to produce acid to maintain vaginal flora balance. In postmenopausal women with recurrent cystitis, low-dose vaginal estrogen therapy controls the growth of pathogens in the vagina (40). Additionally, higher estrogen levels produced by adipose tissue increases the glycogen content in vaginal epithelial cells. The increase in glycogen promotes the colonization of *lactobacilli* and the production of lactic acid, thus supporting a more ideal vaginal environment (41).

Research has also found that progesterone and dehydroepiandrosterone (DHEA) have effects on *lactobacilli*. Progesterone increases the population of *lactobacilli*, affecting brain function by modulating gut microbiota and upregulating the expression of brain-derived neurotrophic factor (BDNF) genes, thereby alleviating depression and anxiety during menopause (42). Additionally, DHEA lowers vaginal pH by stimulating *lactobacilli*, thus relieving symptoms of atrophic vaginitis during menopause (43).

### 3.3 E. coli

*E. coli* is a Gram-negative rod bacterium commonly colonized in the intestines of animals and humans. Most strains are harmless and play important physiological roles in the intestines (44). However, certain strains may be pathogenic, causing various diseases, including sepsis, diarrhea, and urinary tract infections (UTIs) (45).

Among common urinary tract pathogens associated with the development of UTIs, uropathogenic *E. coli* (UPEC) is the main cause (23, 46). The incidence of UTIs significantly increases in postmenopausal women. Decreased estrogen levels may promote the colonization of UPEC in the urinary genital tract and enhance its ability for persistent infection (47–50). UPEC infection is included in a multi-stage infection pathway, including adhesion, invasion, replication, and persistent infection. Studies have shown that estrogen increases the thickness of extracellular glycosaminoglycan (GAG) layer on the bladder surface, affecting the composition and sulfation status of GAG, thereby preventing urinary tract infections caused by *E. coli* (51). However, some studies have found that high-dose estrogen therapy lead to a higher bacterial infection rate of three clinical isolates, type 1, P, and Dr fimbriae of UPEC, in the kidneys (52) (**Figure 1**).

The differences in research results may be related to the “dosage” or pathway of estrogen administration. Additionally, studies have found that under the influence of androgens, both males and females have exhibited increased susceptibility to urinary tract infections caused by UPEC, and activation of the androgen receptor (AR) have increased susceptibility to the formation of pyelonephritis and renal abscesses resulting from *E. coli* infection (53).

The recurrence rate of *E. coli* infection in the urethra is low among women receiving E2 treatment, which is related to the production of antimicrobial peptides (such as human β-defensins HBD-1, HBD-2, ribonuclease (RNase) 7) and the stability of the common microbiota (54). Among postmenopausal women receiving estrogen therapy, an increase in mRNA encoding HBD-1 and HBD-3, psoriasin, and cAMP are observed, thereby reducing the risk of bacterial infection (55).

E2 enhances epithelial integrity by increasing the production of antimicrobial peptides and strengthening intercellular connections, thus promoting epithelial barrier function (55, 56). BD2, also known as β-defensin 2, is a host defense peptide produced by host cells with bactericidal activity against various bacteria, including UPEC. Studies have shown that the concentration of BD2 in the vagina increased in patients receiving estrogen therapy, enhancing bladder innate responses and strengthening urethral epithelial integrity to prevent invasion by pathogenic *E. coli* (57) (**Figure 1**).

E2 also inhibits the production of IL-1β and lipopolysaccharide (LPS)-induced inflammatory effects by *E. coli*. During acute *E. coli* infection, estrogen restricts its proliferation and reduces residual bacteria (55, 56). Lipocalin-2 (Lcn2) is an important molecule that prevents bacterial infection by sequestering iron ions, and *E. coli* induces the expression of Lcn2 in the endometrium. Further research have shown that estrogen induces the expression of Lcn2 in the endometrial epithelium through ERα, thereby enhancing resistance to *E. coli* infection during early pregnancy (58) (**Figure 1**).

*E. coli* may also cause bacteremia, which may lead to sepsis, organ failure, and death when bacteria accumulate and persist in the body (59). Women have lower incidence and mortality rates of *E. coli* bacteremia (4). Studies have shown that colonic pathogenic *E. coli* in the bloodstream is rapidly phagocytosed by Kupffer cells in female mice, while bacterial uptake is delayed in male mice (60). There is a class of natural antibodies against colonic pathogenic *E. coli* in female mouse serum but not in male mouse serum. These antibodies are mainly composed of IgM and IgG3 subtypes, mainly produced by B1 cells, and estrogen indirectly promotes the production of natural antibodies by B1 cells through its effect on peritoneal macrophages (61) (**Figure 1**).

Antibodies against LPS-O127 reduce inflammatory tissue damage and provide life-saving protection in infants. Several research have found that estrogen-mediated interactions between peritoneal macrophages and B1 cells regulate the production of innate antibodies against LPS-O127 (60). Therefore, estrogen-driven natural antibodies against *E. coli* infection play a dual protective role in women and their offspring.

### 3.4 *Pseudomonas aeruginosa* (*P. aeruginosa*)

*P. aeruginosa*, is a ubiquitous Gram-negative bacillus existed in soil, water bodies, plant surfaces, as well as on the surfaces of humans and animals (62). It is a highly drug-resistant pathogen, particularly common in hospital-acquired infections and immunocompromised patients, leading to various infections including respiratory tract infections, urinary tract infections, and wound infections (63).

*P. aeruginosa* is one of the common pathogens causing pulmonary infections in cystic fibrosis (CF) patients, seriously impacting the health of CF patients, resulting in deteriorating lung function, respiratory distress, acute exacerbations, chronic inflammation, and potentially leading to pulmonary fibrosis and airway damage, exacerbating the condition of CF patients (64, 65). Studies have indicated a significant gender difference in cystic fibrosis (CF). Compared to male, the survival rates of female patients are lower and the prognosis is worse (66). Female mice are more susceptible to *P. aeruginosa* infection than male mice, as estrogen and its receptors exacerbate lung inflammation and *P. aeruginosa* infection in CF patients (67). Supplementation of estrogen in male and female mice infected with *P. aeruginosa* reduce bacterial clearance ability (66).

17β-estradiol mediates neutrophil response in the environment of *P. aeruginosa* infection. In ovariectomized mice treated with E2 after *P. aeruginosa* infection, TNF-α, KC, IL-10, and IL-6 significantly increase, along with chemoattractants including granulocyte-macrophage colony-stimulating factor (GM-CSF), monocyte chemoattractant protein-1 (MCP-1), macrophage inflammatory protein-1α (MIP-1α), and MIP-1β (66, 68). Neutrophil granule proteins myeloperoxidase (MPO) and neutrophil elastase (NE) are significantly enriched in lung tissues of mice treated with E2 (66). Furthermore, E2 enhances the production of pro-inflammatory Th17 mediators (IL-23/IL-17 pathway molecules), increases granulocyte colony-stimulating factor (G-CSF) to stimulate the neutrophil production, induces IL-17-mediated chemoattractant factor MIP-2 levels, recruits neutrophils to the site of inflammation, exacerbates the severity of *P. aeruginosa* pneumonia, and promotes lung tissue damage (4, 68). Under gradually increasing E2 doses, the killing ability of neutrophils against *P. aeruginosa* is inhibited (66) (**Figure 1**).

In female CF patients, increased levels of estradiol mediates upregulation of secretory leukocyte protease inhibitor (SLPI) expression via ERβ, thereby inhibiting TLR-dependent IL-8 release in cystic fibrosis bronchial epithelial cells, making female with cystic fibrosis more susceptible to infection and colonization by *P. aeruginosa* (4). E2 also modulates the formation of *P. aeruginosa* biofilms by affecting antimicrobial peptide lactoferrin (LTF), a component of innate immunity that interferes with bacterial biofilm development (68) (**Figure 1**). E2 treatment significantly reduces mRNA levels of antimicrobial peptide LTF in lung tissues and PIP in trachea, with LTF mRNA levels almost completely eliminated after exposure to E2.

Excessive production of alginate in CF patients exacerbates respiratory symptoms and increased the risk of respiratory infections (69). *P. aeruginosa* actively metabolizes steroid hormones and utilized estradiol as a carbon source to promote the production of alginate (70, 71). In menstruating CF females, circulating estradiol levels positively correlates with exacerbations, and increased proportions of the mucoid transition of *P. aeruginosa* are isolates during the period of higher estradiol levels (72). Short-term exposure of *P. aeruginosa* to estradiol results in increased hydrogen peroxide levels, and inhibits hydrogen peroxide detoxifying enzyme activity. Estradiol promotes mucoid transition of *P. aeruginosa*, increases alginate production and selectively induces mucA gene mutations (a negative regulator of alginate synthesis), which is due to impaired catalase activity and increased hydrogen peroxide levels (72).Therefore, female CF patients are infected with *P. aeruginosa* earlier than males, transitioning to mucoid strains prematurely, exacerbating respiratory symptoms, and making infections more difficult to clear (72).

The incidence of bacterial pneumonia is high in patients with severe traumatic brain injury (TBI) (73), with *P. aeruginosa* being one of the most common pathogens (74). Studies have found that *P. aeruginosa* pneumonia after TBI lead to higher mortality and reduced bacterial clearance, and gender differences are observed. Compared to female mice, male mice have higher mortality rates (75). There are differences between male and female macrophages, with female macrophages secreting higher levels of TNF-α compared to male macrophages (75). Severe TBI patients are at higher risk of bacterial pneumonia due to immune suppression. The administration of estradiol reduces mortality in male or ovariectomized wild-type female mice, and increases lung bacterial clearance, possibly mediated by differences in alveolar macrophage function (**Figure 1**).

The role of estrogen in *P. aeruginosa* infection varies depending on the type of disease, possibly related to estrogen dose, route of administration, and mediated signaling pathways.

### 3.5 Staphylococcus aureus

*S. aureus* is a prevalent Gram-positive bacterium found on the human skin and mucous membranes. Although it is usually harmless, *S. aureus* could also cause various infections, including skin infections, food poisoning, respiratory tract infections, and sepsis (76).

As a major pathogen of global skin and soft tissue infections (SSTIs), the toxin alpha-hemolysin (Hlα) produced by *S. aureus* is a key virulence factor causing skin necrosis and inflammation, promoting invasive infections by destroying cell junctions (77–79). Studies have shown that males are more susceptible to SSTIs caused by *S. aureus* compared to females (80) (**Figure 1**). Furthermore, research have found that estrogen plays an important role in regulating the innate immune response of females to *S. aureus* pathogens (14, 80). Female mice exhibit lower levels of pro-inflammatory cytokines such as IL-1β, TNFα, IL-6, and CXCL1 at the site of *S. aureus* infection, along with stronger neutrophil bactericidal activity (80). Female mice show estrogen-dependent reduction in skin necrosis, lower levels of local inflammatory cytokines, and a significantly reduced bacterial burden after infection. Additionally, in the rat uterus, E2 induces antimicrobial peptides to inhibit the invasion of *S. aureus* (55) (**Figure 1**).

G-1, a highly selective ligand of GPER, has been shown to reduce the expression of the Hlα receptor ADAM10 on the surface of keratinocytes by modulating GPER, thereby limiting the disruption of epithelial barrier integrity mediated by Hlα, further alleviating the severity of *S. aureus*-induced SSTIs. In the mouse infection model, G-1 reduces the production of pro-inflammatory cytokines and enhances the host innate immune defense against major bacterial toxins (81) (**Figure 1**).

Nasal carriage of *S. aureus* is the most important reservoir of this pathogen, with significant implications for pathogen transmission and infection (82). Research has found that women taking hormonal contraceptives are more likely to become continuous carriers of *S. aureus* in the nose, and reproductive hormone intake is correlated with the persistence of *S. aureus* nasal colonization (83). However, this study does not specifically differentiate between estrogen and progesterone contraceptives for an in-depth investigation. While physiological concentrations of progesterone has been found to inhibit the growth of Staphylococcus aureus, the specific role of estrogen in nasal colonization by *S. aureus* remains unclear (84).

As an opportunistic bacterium, *S. aureus* may also cause anaerobic vaginitis (85). Studies suggest that ERα may be a key factor in *S. aureus* -induced vaginal infections. A certain dose of E2 may promote the adhesion of *S. aureus* to human vaginal epithelial cells through the ERα/FAK/Src/iNOS axis, thereby accelerating *S. aureus* infection of the vagina (86) (**Figure 1**).

Previous studies have shown that estrogen has a dual effect on diseases caused by *S. aureus* infection, and the specific role of estrogen should be determined based on the type of disease. Therefore, estrogen can be used as an intervention point to take corresponding preventive and therapeutic measures to prevent and slow down disease progression.

### 3.6 Helicobacter pylori

*H. pylori* is a Gram-negative spiral-shaped bacterium commonly found on the mucous membranes of the human stomach (87), which is considered to be the major causative factor for gastric ulcer disease and gastric cancer (88).According to epidemiological data, the incidence of gastric cancer in humans is predominantly higher in males, with a male-to-female ratio of 2:1 (89).

In *H. pylori*-induced gastric injury, pro-inflammatory Th1 cytokines including IFN-γ, IL-1β, and TNF-α, along with Th17 cells, participates in the pathogenesis (90, 91). Foxp3-positive regulatory T cells and pro-inflammatory Th17 cells play crucial roles in the development of *H. pylori*-induced gastric inflammation in mice. Studies have indicated that E2 treatment in male INS-GAS mice infected with *H. pylori* can reduce mRNA levels of IFN-γ and IL-1β by upregulating gastric IL-10 activity and increase mRNA expression of Foxp3 (92). Furthermore, E2 supplementation also reduces gastric epithelial cell proliferation in both *H. pylori*-infected and uninfected mice (92, 93). Treatment with E2 after *H. pylori* infection trigger a Th2-skewed cytokine profile in the gastric mucosa while alleviating gastric pathology (93) (**Figure 1**). Studies have also shown that the female sex hormone progesterone can mediate anti-inflammatory responses, inhibiting the growth of *H. pylori* and exerting a protective effect (94, 95).

GPER, a membrane estrogen receptor, mediates rapid non-genomic estrogen actions by binding with estrogen on the cell membrane (96). Cytotoxin-associated gene A (CagA) is one of the major virulent factors produced by *H. pylori* (97). IL-8 is a typical inflammatory cytokine upregulated in *H. pylori* infection and correlates with the histological severity of damaged gastric mucosa (98, 99).The activation of the CagA-mediated signaling pathway promotes the expression of the inflammatory cytokine IL-8 via the NF-κB pathway, leading to chronic gastritis and peptic ulcers (100) (**Figure 1**). Studies have shown that GPER can downregulate IL-8 expression in gastric cell lines by inhibiting NF-κB promoter activation induced by CagA expression, thereby mitigating inflammation and gastric mucosal damage (100) (**Figure 1**).

Estrogen-related receptor gamma (ER-γ) is a nuclear receptor identified as a tumor suppressor gene in several cancers, particularly sex-related tumors (101). In gastric cancer, ER-γ binds to the Trefoil factor 1(TFF1) promoter and induces TFF1 gene expression (102, 103). TFF1, a tumor suppressor, is a downstream target of ER-γ, and *H. pylori* infection downregulates ER-γ expression. NF-κB is an inflammatory transcription regulator, and the enhanced phosphorylation of NF-κB/p65 occurs in *H. pylori* infection and its related CagA pathogenic protein (104). Research has found that ER-γ protects gastric cells from *H. pylori* infection and inhibits gastric cancer cell growth by regulating TFF1 and NF-κB (105) (**Figure 1**).

*H. pylori* infection is also a risk factor for diffuse gastric cancer (DGC), with higher mortality rates observed in young female DGC patients (106, 107). Studies have found that estrogen affects DGC organoids and directly regulates the oncogenic HOTAIR, promoting tumorigenesis in the presence of ERα (108, 109) (**Figure 1**). Estrogen-dependent activation of HOTAIR can occur in various forms, and the Mixed Lineage Leukemia factor (MLL) family is a known ER co-regulator. MLL1 and MLL3 bind to the promoter of the HOTAIR gene in the presence of 17β-estradiol (110). In ERα-positive Diffuse Gastric Cancer (DGC), *H. pylori* secretes CagA, which recruits the co-regulatory factor MLL3 to promote HOTAIR transcription. Consequently, estrogen binds to the Estrogen Response Element on the HOTAIR promoter, thereby enhancing the expression of oncogenic HOTAIR. This leads to the induction of epithelial-mesenchymal transition (EMT) and stem cell phenotypes in both diffuse gastric cancer cells and organoids, thereby accelerating the progression of diffuse gastric cancer (109).

*H. pylori* also promotes the occurrence and progression of cholangiocarcinoma. *In vitro* observation of human intrahepatic biliary epithelial cells (HIBECs) reveals that *H. pylori* and 17β-estradiol, either alone or in combination, promote HIBEC proliferation, inhibit apoptosis, and induce a certain degree of invasiveness (111) (**Figure 1**). In general, estrogen plays different roles in diseases caused by *H. pylori* infection.

### 3.7 Non-tuberculous Mycobacteria and Mycobacterium tuberculosis

NTM are a group of Gram-positive rods widely distributed in soil, natural water, and artificial aquatic systems. Humans are often exposed to NTM, the majority of which are non-pathogenic, but some strains could cause human diseases (112, 113).

**Figure 2:**
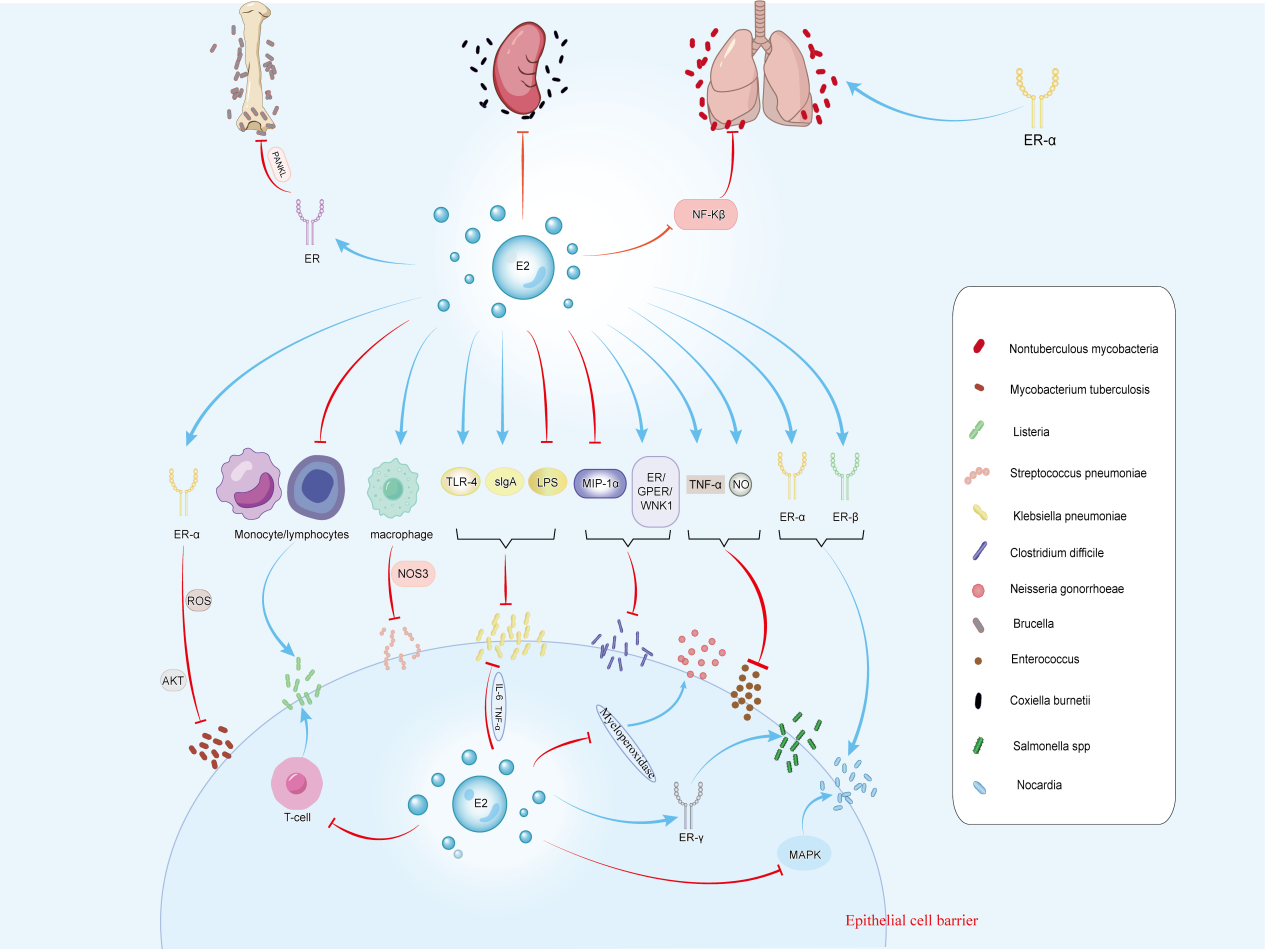
The figure illustrates the effects of estrogen on 12 types of *bacteria*, including *M. tuberculosis*, *Listeria*, *S. pneumoniae*, *Klebsiella*, and *C. difficile*. Estrogen can regulate bacterial infections through receptors and related signaling pathways. It inhibits infections caused by *Klebsiella*, *C. difficile*, *S. pneumoniae*, *Enterococcus*, *Brucella*, *M. tuberculosis*, and *C. burnetii*. Conversely, it promotes infections by *Listeria*, *N. gonorrhoeae*, *Salmonella*, and *Nocardia*. For *non-tuberculous mycobacterial* infections, estrogen has dual effects.

NTM infections lead to chronic pulmonary diseases, with NTM lung diseases becoming increasingly common among postmenopausal women with slender body shapes (114). Studies have suggested that postmenopausal women with NTM lung diseases show estrogen imbalance. Additionally, experimental evidence suggests that estradiol can inhibit the activation of the transcription factor NF-κB in mouse macrophages (115) (**Figure 2**). NF-κB inhibition results in apoptosis of macrophages infected with *M. tuberculosis*, which is a well-known mechanism of *M. tuberculosis* skilling. Thus, estrogen may enhance its killing effect by promoting the intracellular mycobacterial apoptosis (114, 116).

Lady Windermere syndrome, characterized by low body mass index (BMI), tall stature, and a high prevalence of kyphoscoliosis, pectus excavatum, and mitral valve prolapse, is associated with NTM pulmonary infections (117). It is believed that estrogen deficiency leads to immune response disorders and other aspects of NTM, resulting in this syndrome (118). Moreover, the relative leptin deficiency in lean individuals can lead to decreased estrogen levels, which is another potential risk factor for NTM infections (114).

The prevalence and burden of nontuberculous mycobacterial pulmonary disease (NTM-PD) are increasing globally (119). The duration of hormone replacement therapy is positively associated with the risk of NTM-PD in postmenopausal women (120). Furthermore, research has found the regulatory effect of estrogen receptors. In *M. tuberculosis* infection, ER-α-deficient mice produce more infection-controlling cytokines, namely IL-18 and IFN-γ (121).

*M. tuberculosis* is the causative agent of tuberculosis (122), which can enter the human body through droplet transmission, causing pulmonary tuberculosis, or invading other organs, leading to other forms of tuberculosis, such as lymph node tuberculosis and skeletal tuberculosis (123). After being engulfed by host cells*, M. tuberculosis* replicates within the infected cells and prevents the elimination of target bacteria by inhibiting the maturation of phagosomes. Host cells control this evasion mechanism by inducing autophagy, a complex cellular process that gradually eliminated bacteria and reduce the bacterial load within infected cells (124). Studies have shown that ER-α is a transcriptional activator of autophagy genes, promoting the initiation and execution of autophagy by regulating the expression of autophagy-related genes, thereby enhancing the host’s defense against intracellular pathogens (125). In the context of tracheobronchial tuberculosis infection, estradiol primarily acts through ERα binding, affecting ROS generation and the AKT signaling pathway, thereby inhibiting autophagy in *M. tuberculosis-*infected cells and controlling the proliferation of intracellular *M. tuberculosis (* 126) (**Figure 2**).

### 3.8 Sepsis

Sepsis is a severe systemic inflammatory response, typically caused by bacterial infection (127). When the body responds to the infection, the immune system releases a large amount of chemicals into the bloodstream, initiating an inflammatory response which leads to systemic symptoms such as fever, accelerated heart rate, shortness of breath, and lower blood pressure (128, 129).

Clinical studies have shown a higher incidence of sepsis in males compared to age-matched female populations (130). Research has indicated that agonists of ER-β provide significant survival advantages in experimental models of bacterial sepsis, while reducing tissue damage and decreasing levels of various pro-inflammatory proteins (131). ER-β is present in intestinal epithelial cells, and the gastrointestinal mucosa is a major source of microbiota-derived mediators released systemically during sepsis (132). ER-β agonists can reduce the level of circulating endotoxin and peritonitis cytokines, decrease the number of intestinal bacteria in the bloodstream after cecal ligation and puncture in mice, thereby improving the results of experimental systemic infections, and maintaining the function and structure of the gastrointestinal mucosa (132, 133).

Heat shock protein 70 (HSP70) is a molecular chaperone involved in heat stress, promoting protein folding and preventing the secretion of inflammatory mediators, thereby reducing the mortality of sepsis (134, 135). Modulation of the heme oxygenase-1 (HO-1) pathway can offset oxidative stress, improve myocardial function in endotoxemia, and increase survival rates in septic mice (136). Studies have proved that estrogen receptor modulators upregulated HSP70 and HO-1 by mediating ER-α activation, exerting antioxidant and anti-inflammatory effects, and alleviating the severity of sepsis (137).

In septic infections, serum haptoglobin (Hp) plays a balanced role in maintaining the cascade of pro-inflammatory and anti-inflammatory responses. Estrogen can regulate the increase in Hp levels, while Hp maintains endotoxin tolerance by reducing TNFα levels (the main mediator of cytokine storm), thereby slowing the progression of bacterial sepsis (138).

In addition to the protective effects of estrogen in sepsis, progesterone can also improve the sepsis syndrome by reducing the levels of inflammatory factors IL-6 and TNF-α, and by restoring antioxidant enzyme activity in some tissues (139).

### 3.9 Other bacterial infections

*Listeria*: *Listeria monocytogenes* (*L. monocytogenes*) is a Gram-positive bacterium commonly found in soil, water, and animal feces. It can cause foodborne infections, and for pregnant women, infection with *L. monocytogenes* may result in miscarriage, stillbirth, or neonatal infection (140). Studies have shown a direct correlation between estrogen activity and increased susceptibility to *L. monocytogenes* infection. Estrogen exposure inhibits the accumulation of lymphocytes and monocytes in the peritoneal cavity of mice, possibly mediates through the estrogen receptor mechanism, partly via thymus regulation, and ultimately suppresses the activation of T-cell-dependent defense mechanisms, thereby exacerbating the infection (141) (**Figure 2**). Additionally, estrogen may reduce the proliferation of antigen-sensitive T lymphocytes by inhibiting IL-2 production, thereby weakening the host’s resistance to *L. monocytogenes* (142).

*Streptococcus pneumoniae*: *Streptococcus pneumoniae* (*S. pneumoniae*) is a Gram-positive bacterium commonly present in the human upper respiratory tract and oropharynx, capable of causing various diseases including pneumonia, otitis media, meningitis, and sepsis (143).Female mice exhibit stronger resistance to *S. pneumoniae*, with research indicating that estrogen mediates this enhanced host resistance by affecting NOS3 in pulmonary macrophages of female mice (144) (**Figure 2**). In pneumococcal meningitis caused by *S. pneumoniae* infection, ER-β influences microglial cell status by downregulating the NF-κB signaling pathway, promoting neuronal recovery through wound healing mechanisms (145–147). The activation of ER-β also induces the expression of brain-derived neurotrophic factor, a neuroprotective factor that promotes the survival of local neurons after injury (146).

*Klebsiella pneumoniae*: *Klebsiella pneumoniae* (*K. pneumoniae*) is a Gram-negative bacillus commonly found in the respiratory tract and can cause pneumonia (148). Research suggest that E2 enhances the transport of sIgA to the respiratory tract and increases TLR-4 expression, thereby helping to prevent *K. pneumoniae*-infected pneumonia (149) (**Figure 2**).

*K. pneumoniae* is also known as a pathogen colonizing on the skin (150). LPS delays several aspects of wound healing, inducing excessive cell death, stalling keratinocyte activation, enhancing local inflammation, and reducing collagen deposition (151). Studies suggest that estrogen weakens LPS-induced inflammatory signals through IL6 and TNFα, thereby reducing macrophage infiltration and improving wound closure and delays epidermal regeneration (151) (**Figure 2**).

*Clostridium difficile*: *Clostridium difficile* (*C. difficile*) is a Gram-positive bacterium commonly found in soil, water, and feces. It forms heat-resistant spores, which can cause *C. difficile* infection (CDI), leading to gastrointestinal inflammation and symptoms such as diarrhea, abdominal pain, and fever (152, 153). Macrophage inflammatory protein 1α (MIP-1α) mediates CDI, which typically results in epithelial damage and neutrophil infiltration in the colonic mucosa of infected mice (154). Studies have shown that soy isoflavones, acting as estrogen-like compounds, reduces mortality in infected hamsters by inducing an anti-apoptotic effect in epithelial cells mediated by ER/GPER/WNK1 and inhibiting MIP-1α expression, thereby improving cecal damage and CDI disease activity and providing protective effects against *C. difficile* toxins (154, 155) (**Figure 2**).

*N. gonorrhoeae*: *N. gonorrhoeae*, a Gram-negative bacterium, is the pathogen of gonorrhea, which is most commonly manifested by male urethritis and female cervicitis or urethritis (143). Animal studies have indicated that female mice are most susceptible to *N. gonorrhoeae* infection in the early stage of estrus. The treatment of estradiol affected the bactericidal activity of polymorphonuclear leukocytes mediates by myeloperoxidase, enhancing the susceptibility of mice to disseminated gonococcal infection (23, 156) (**Figure 2**).

Gonococcal vaginitis is the most common form of *N. gonorrhoeae* infection in children after the neonatal period. Compared to adolescents and adults, due to the low level of estrogen, the vaginal environment of prepubertal children is relatively normal to alkaline pH, with a thinner layer of vaginal mucosal cells, making it easier for *N. gonorrhoeae* to infect and colonize the vagina (157).

*Brucella*: *Brucella* is a genus of Gram-negative bacteria that can cause brucellosis, which is a zoonotic disease. The bacteria are primarily present in mammals such as cattle, sheep, and pigs, and are transmitted to humans through contact with infected animals (158).

Osteoarticular brucellosis is the most common presentation of active brucellosis in humans, where *Brucella* spp. directly or indirectly impaired osteoblast function and induced osteoblast apoptosis. Furthermore, *Brucella* infection lead to the occurrence of pathological osteoclasts, resulting in damage to the bones and joints of patients with osteoarticular brucellosis (159). Studies have shown that *Brucella* infection inhibited extracellular matrix deposition by osteoblasts, while DHEA treatment can reverse this response via estrogen receptor signaling (160). Additionally, *Brucella* infection of human synovial cells induces increased expression of RANKL. Overexpression of RANKL in pathological conditions may lead to osteoporosis, fractures, and other skeletal diseases. Research suggests that ER regulates osteoclast formation in Brucella-infected synovial cells by modulating the key molecule RANKL (161) (**Figure 2**). Furthermore, low estradiol levels are also an important risk factor for osteoporosis (162).

*Enterococcus*: *Enterococcus* is a genus of Gram-positive cocci and is commonly found in the intestines of both animals and humans. *Enterococcus* causes healthcare-associated infections, especially in patients who are already debilitated or immunocompromised (163). Estradiol administration prevents bacteremia following intraperitoneal inoculation of *Enterococcus* in ovariectomized rats by increasing TNF-α and NO levels (4) (**Figure 2**).

*Coxiella burnetii*: *Coxiella burnetii* (*C. burnetii*), the pathogen of Q fever, is a Gram-negative bacterium (164). Q fever is a zoonotic infectious disease caused by *C. burnetii*, typically transmitted to humans via respiratory tract or contact with infected sources. Symptoms of Q fever usually include high fever, headache, muscle pain, cough, and complications such as pneumonia, hepatitis, and pericarditis may occur in severe cases (4) (**Figure 2**).

Studies have shown that in female mice infected with *C. burnetii*, the number of granulomas in the spleen is lower compared to that of male mice infected with *C. burnetii*, and treatment with 17β-estradiol can reduce *C. burnetii* load and prevent the upregulation of granuloma formation (165).

*Salmonella* spp.: *Salmonella* is a genus of Gram-negative rods and is a common pathogen, causing various diseases such as food poisoning and gastrointestinal infections (166).

Research indicates a significant association between estrogen therapy and *Salmonella* infection. Estradiol-treated mice are more susceptible to typhoid *Salmonella* than the control group, with a higher mortality rate (167). Additionally, estrogen exposure weakens the antibacterial effect of *Salmonella* in the abdominal cavity (168).

Studies have found that *S. typhimurium* induces hepcidin expression, hypo-ferraemia, and ERRγ expression in mice. ERRγ acts on the downstream of IL-6 and negatively impacts host defense against infection (169) (**Figure 2**). Furthermore, overexpression of ERRγ induces hepcidin production and hypoferraemia, while ERRγ inverse agonists can reduce its activity. These inverse agonists improve *Salmonella*-induced hepcidin expression, hypo-ferraemia, and hepatic and splenic iron accumulation, while reducing bacterial load, macrophage iron content, and pro-inflammatory cytokine production, thereby enhancing host survival rates (169, 170).

*Nocardia: Nocardia* is a type of Gram-positive bacteria that is typically found in soil, water, and decaying organic matter (171). In medicine, *Nocardia* infections are often associated with respiratory infections, skin infections, and infections in other organs, often requiring long-term antibiotic treatment (172). Studies have found that female mice infected with *Nocardia* have a higher mortality rate. E2 is found to impair the ability of mice to clear Nocardia, as it can bind to ER-α and ER-β to facilitate the entry of *Nocardia* into host cells, leading to severe cellular damage. Additionally, E2 is observed to promote bacterial survival by inhibiting the inflammatory response mediated by the MAPK pathway (173) (**Figure 2**).

Disseminated odontogenic infections results from a large number of bacteria entering surrounding tissues from the root canal system, evading and disrupting local immune mechanisms. Bacterial dissemination lead to serious complications such as sinusitis, airway obstruction, cavernous sinus thrombosis, brain abscess, and even death (174). Studies suggest that females may have higher resistance to the spread of odontogenic infections compared to males. IL-1 promotes the activity of neutrophils and monocytes, enhancing their antimicrobial activity. Estradiol stimulates and activates neutrophils by increasing IL-1 expression, constituting a critical host defense mechanism against the spread of odontogenic infections (175).

## 4 Conclusion

The difference in estrogen levels between men and women may be a key factor in determining different outcomes after bacterial infections. Estrogen influences the regulatory role of the immune system against bacteria. In some bacterial infections, such as SSTI induced by *S. aureus*, gastric cancer caused by *H. pylori* infection, and bacterial infections caused by lung diseases induced by NTM, males tend to exhibit higher susceptibility. Additionally, estrogen can mediate the progression of diseases through estrogen receptor signaling pathways. For example, in *C. trachomatis* infection, antibodies against ERα and/or ERβ can reduce the infectivity of *Chlamydia*. During early pregnancy, ERα enhances resistance to *E. coli* infections. Furthermore, ERβ agonists lowers circulating endotoxin levels and pro-inflammatory cytokines, reducing the number of intestinal bacteria entering the bloodstream. Modulating GPER alleviates the severity of skin and soft tissue infections caused by *S. aureus* and mitigates the inflammatory response and gastric mucosal damage caused by *H. pylori*.

Extensive clinical studies have demonstrated the therapeutic potential of exogenous estrogen treatment. Administering exogenous estrogen or estrogen receptor agonists is necessary for diseases in which estrogen plays a beneficial role in bacterial infections. The availability of these treatment modalities provides important avenues for improving patient prognosis. Conversely, estrogen receptor antagonists, which mediated pro-inflammatory responses, may have beneficial therapeutic effects.

For different types of bacterial infections, individualized treatment plans for different gender and estrogen levels may be required. By comprehensively considering the biological effects of estrogen and clinical research findings, we can better understand the role of estrogen in bacterial infections and develop more effective prevention and treatment strategies to improve the prognosis and quality of life for patients.
