# Supplementary material for: Estrogen and bacterial infection
Source: Front Immunol. 2025 Apr 29;16:1556683. doi: 10.3389/fimmu.2025.1556683 (PMC12069284; doi:10.3389/fimmu.2025.1556683)
Supplement: Supplementary file 1 [file DataSheet1.pdf]

Supplementary Material

Estrogen and bacterial infection

Longyan Hong, Hao Liang
*** Correspondence:** Pengbo Guo:877273621@qq.com

1 Supplementary Figures and Tables

Table1: The Impact of Estrogen on Select Gram-positive bacteria.

	Bacterial type	Disease caused	The effect of estrogen or estradiol and its receptors	Susceptibility or prognosis
Gram-positive bacteria	*Lactobacillus*	Microorganism of normal vaginal flora	Estrogen could promote the proliferation of lactobacilli, which could inhibit the growth of pathogens[1, 2].	—
	Staphylococcus aureus	Skin infections<br>Food poisoning<br>Respiratory tract infections<br>Sepsis<br>Global skin and soft tissue infections (SSTIs)[3]	Estrogen and its receptors reduce the production of pro-inflammatory cytokines and enhance the host's immune defense against major bacterial toxins[4, 5].	Men are more susceptible
		Anaerobic vaginitis [6]	E2 could promote the adhesion of *S. aureus* to human vaginal epithelial cells through the ERα/FAK/Src/iNOS axis, thereby accelerating *S. aureus* infection of the vagina [7].	Women
	Nontuberculous mycobacteria	Chronic pulmonary diseases[8]	Estrogen might enhance its killing effect by promoting the intracellular *mycobacterial* apoptosis[9, 10].	Postmenopausal women are more susceptible
		Nontuberculous mycobacterial pulmonary disease	Estrogen and ER- α may promote the infection of non-tuberculous *mycobacterial* pulmonary disease[11].	Women are more susceptible.
	Mycobacterium tuberculosis	Pulmonary tuberculosis<br>Lymph node tuberculosis<br>Skeletal tuberculosis [12]	Estradiol could inhibit autophagy in *Mycobacterium tuberculosis*-infected cells and control the proliferation of intracellular *Mycobacterium tuberculosis*[13].	The prognosis of male patients is poor.
	Listeria monocytogenes	Foodborne infections<br>Miscarriage<br>Stillbirth<br>Neonatal infection[14]	Estrogen activity exacerbates *Listeria monocytogenes* infection by weakening the host's resistance to the bacterium[15].	Women are more susceptible.
	Streptococcus pneumoniae	Pneumonia<br>Otitis media<br>Meningitis<br>Sepsis [16]	Estrogen and its receptor β can attenuate the onset and progression of diseases caused by *Streptococcus pneumoniae*[17-19].	Men are more susceptible.
	Clostridium difficile	Gastrointestinal inflammation<br>Diarrhea<br>Abdominal pain<br>Fever[20]	Estrogen-like compounds could reduce mortality in infected hamsters, and improve cecal damage and CDI disease activity and provide protective effects against *C. difficile* toxins[20, 21].	The prognosis of male patients is poor.
	Enterococcus	Healthcare-associated infections	Estradiol could prevent bacteremia following intraperitoneal inoculation of *Enterococcus* in ovariectomized rats by increasing TNF-α and NO levels[22].	The debilitated or immunocompromised patients are more susceptible.
	Nocardia	Respiratory infections<br>Skin infections<br>infections in other organs[23]	Estrogen and its receptors can facilitate the entry of *Nocardia* into host cells, resulting in severe cellular damage[24].	Women are more susceptible.

Table2: The Impact of Estrogen on Select Gram-negative bacteria.

	Bacterial type	Disease caused	The effect of estrogen or estradiol and its receptors	Susceptibility or prognosis
Gram-negative bacteria	*Chlamydia trachomatis*	Urethritis<br>Cervicitis<br>Pelvic inflammatory disease [25]	Estrogen and its receptors increase the risk of *Chlamydia trachomatis* infection[26].	Women are more susceptible.
	Escherichia coli	Sepsis<br>Diarrhea<br>Urinary tract infections	Estrogen could prevent urinary tract infections caused by *Escherichia coli*[27, 28].<br>E2 could inhibit the production of IL-1β and lipopolysaccharide (LPS)-induced inflammatory effects by *Escherichia coli*[27, 28].<br>Estrogen could restrict *Escherichia coli* proliferation and reduce residual bacteria [29].<br>Estrogen could indirectly promote the production of natural antibodies by B1 cells through its effect on peritoneal macrophages[30].	Men are more susceptible.
	Pseudomonas aeruginosa	Respiratory tract infections<br>Urinary tract infections<br>Wound infections<br>Pulmonary infections in cystic fibrosis (CF) patients [31].	Estrogen and its receptors could exacerbate lung inflammation and *P. aeruginosa* infection, reduce bacterial clearance ability, and inhibit the killing ability of neutrophil against *P. aeruginosa*.<br>Estradiol could make *P. aeruginosa* more susceptible to infection and colonization [32, 33].	The survival rates of female CF patients are lower<br>and the prognosis is worse
		Bacterial pneumonia in TBI patients [34].	Estradiol could reduce mortality and increase lung bacterial clearance [35].	The survival rates of male TBI patients with bacterial pneumonia are lower.
	Helicobacter pylori	Gastric ulcer disease. Gastric cancer[36].	Estrogen and its receptors can alleviate inflammation and gastric mucosal damage caused by *H. pylori* infection and inhibit the growth of gastric cancer cells[37, 38].	The prognosis of male patients is poor.
		Diffuse gastric cancer (DGC)[39]	Estrogen could affect DGC organoids and directly regulate the oncogenic HOTAIR, promoting tumorigenesis in the presence of ERα [40].	The mortality rates in young female DGC patients are higher.
		Cholangiocarcinoma [41]	E2 promoted HIBEC proliferation, inhibited apoptosis, and induced a certain degree of invasiveness[42].	Women are more susceptible.
	Klebsiella pneumoniae	Pneumonia[43]	Estrogen can ameliorate diseases caused by *Klebsiella pneumoniae* infection[44].	Men are more susceptible.
	Neisseria gonorrhoeae	Male urethritis<br>Female cervicitis<br>Female urethritis[45]	Estradiol could affect the bactericidal activity of polymorphonuclear leukocytes mediated by myeloperoxidase, and enhance the susceptibility of mice to disseminated gonococcal infection[46, 47].	Women are more susceptible.
		Gonococcal vaginitis [48]	Due to the low level of estrogen, the layer of vaginal mucosal cells of prepubertal children is relatively thinner, making it easier for *N. gonorrhoeae* to infect and colonize the vagina [24].	The prepubertal children are more susceptible.
	Brucella	Brucellosis<br>osteoarticular Brucellosis [49]	*Brucella* infection inhibited extracellular matrix deposition by osteoblasts, while DHEA treatment could reverse this response via estrogen receptor signaling [50].<br>ER could regulate osteoclast formation in *Brucella*-infected synovial cells by modulating the key molecule RANKL [51, 52].	The prognosis of male patients is poor.
	Coxiella burnetii	Q fever[53]	17β-estradiol could reduce *C. burnetii* load and prevent the upregulation of granuloma formation[54].	The prognosis of male patients is poor.
	Salmonella spp.	Food poisoning<br>Gastrointestinal infections [55]	Estrogen and its related receptor ERRγ can promote *Salmonella* infection [56].	The prognosis of female patients is poor.

REFERENCES

1. Thomas-White K, Taege S, Limeira R *et al*: **Vaginal estrogen therapy is associated with increased Lactobacillus in the urine of postmenopausal women with overactive bladder symptoms**. *American journal of obstetrics and gynecology* 2020, **223**(5):727.e721-727.e711.
2. Amabebe E, Anumba DOC: **The Vaginal Microenvironment: The Physiologic Role of Lactobacilli**. *Frontiers in medicine* 2018, **5**:181.
3. Del Giudice P: **Skin Infections Caused by Staphylococcus aureus**. *Acta dermato-venereologica* 2020, **100**(9):adv00110.
4. Kovats S: **Estrogen receptors regulate innate immune cells and signaling pathways**. *Cellular immunology* 2015, **294**(2):63-69.
5. Harding AT, Heaton NS: **The Impact of Estrogens and Their Receptors on Immunity and Inflammation during Infection**. *Cancers* 2022, **14**(4).
6. Donders GG, Ruban K, Bellen G: **Selecting anti-microbial treatment of aerobic vaginitis**. *Current infectious disease reports* 2015, **17**(5):477.
7. Yan L, Rui C, Zhuang B *et al*: **17β-Estradiol Mediates Staphylococcus aureus Adhesion in Vaginal Epithelial Cells via Estrogen Receptor α-Associated Signaling Pathway**. *Current Microbiology* 2023, **80**(12):391.
8. Bents SJ, Mercaldo RA, Powell C *et al*: **Nontuberculous mycobacterial pulmonary disease (NTM PD) incidence trends in the United States, 2010-2019**. *BMC infectious diseases* 2024, **24**(1):1094.
9. Chan ED, Iseman MD: **Slender, Older Women Appear to Be More Susceptible to Nontuberculous Mycobacterial Lung Disease**. *Gender Medicine* 2010, **7**(1):5-18.
10. Kim SY, Yang CS, Lee HM *et al*: **ESRRA (estrogen-related receptor α) is a key coordinator of transcriptional and post-translational activation of autophagy to promote innate host defense**. *Autophagy* 2018, **14**(1):152-168.
11. Choi H, Han K, Yang B *et al*: **Female Reproductive Factors and Incidence of Nontuberculous Mycobacterial Pulmonary Disease Among Postmenopausal Women in Korea**. *Clinical infectious diseases : an official publication of the Infectious Diseases Society of America* 2022, **75**(8):1397-1404.
12. Kalsdorf B, Lange C: **Tuberculous mediastinal lymphadenopathy: Reaching the target**. *Respirology (Carlton, Vic)* 2019, **24**(7):622-623.
13. Gan Y, Hu Q, Li A *et al*: **Estradiol inhibits autophagy of Mycobacterium tuberculosis-infected 16HBE cells and controls the proliferation of intracellular Mycobacterium tuberculosis**. *Molecular medicine reports* 2022, **25**(6).
14. Radoshevich L, Cossart P: **Listeria monocytogenes: towards a complete picture of its physiology and pathogenesis**. *Nature Reviews Microbiology* 2018, **16**(1):32-46.
15. Pung OJ, Tucker AN, Vore SJ *et al*: **Influence of estrogen on host resistance: increased susceptibility of mice to Listeria monocytogenes correlates with depressed production of interleukin 2**. *Infection and immunity* 1985, **50**(1):91-96.
16. Narciso AR, Dookie R, Nannapaneni P *et al*: **Streptococcus pneumoniae epidemiology, pathogenesis and control**. *Nature Reviews Microbiology* 2025, **23**(4):256-271.
17. Lee S, Lee SO, Kim GL *et al*: **Estrogen receptor-beta of microglia underlies sexual differentiation of neuronal protection via ginsenosides in mice brain**. *CNS Neurosci Ther* 2018, **24**(10):930-939.
18. Meltser I, Tahera Y, Simpson E *et al*: **Estrogen receptor beta protects against acoustic trauma in mice**. *The Journal of clinical investigation* 2008, **118**(4):1563-1570.
19. Colonna M, Butovsky O: **Microglia Function in the Central Nervous System During Health and Neurodegeneration**. *Annual review of immunology* 2017, **35**:441-468.
20. Xie Y, Fontenot L, Estrada AC *et al*: **Genistein Inhibits Clostridioides difficile Infection via Estrogen Receptors and Lysine-Deficient Protein Kinase 1**. *J Infect Dis* 2023, **227**(6):806-819.
21. Zhou T, Meng C, He P: **Soy Isoflavones and their Effects on Xenobiotic Metabolism**. *Current drug metabolism* 2019, **20**(1):46-53.
22. Saia RS, Garcia FM, Cárnio EC: **Estradiol protects female rats against sepsis induced by Enterococcus faecalis improving leukocyte bactericidal activity**. *Steroids* 2015, **102**:17-26.
23. Kandi V: **Human Nocardia Infections: A Review of Pulmonary Nocardiosis**. *Cureus* 2015, **7**(8):e304.
24. Han L, Ji X, Liu X *et al*: **Estradiol Aggravate Nocardia farcinica Infections in Mice**. *Frontiers in immunology* 2022, **13**:858609.
25. Ladany S, Sarov I: **Recent advances in Chlamydia trachomatis**. *European journal of epidemiology* 1985, **1**(4):235-256.
26. Li N, Xiao C, Li Y *et al*: **Association of Chlamydia trachomatis Infection With Breast Cancer Risk and the Modification Effect of IL-12**. *Clinical Breast Cancer* 2024, **24**(7):e554-e559.e551.
27. Medina-Estrada I, Alva-Murillo N, Lopez-Meza JE *et al*: **Immunomodulatory Effects of 17beta-Estradiol on Epithelial Cells during Bacterial Infections**. *J Immunol Res* 2018, **2018**:6098961.
28. Luthje P, Brauner H, Ramos NL *et al*: **Estrogen supports urothelial defense mechanisms**. *Sci Transl Med* 2013, **5**(190):190ra180.
29. Engelsöy U, Svensson MA, Demirel I: **Estradiol Alters the Virulence Traits of Uropathogenic Escherichia coli**. *Frontiers in microbiology* 2021, **12**:682626.
30. Zeng Z, Surewaard BGJ, Wong CHY *et al*: **Sex-hormone-driven innate antibodies protect females and infants against EPEC infection**. *Nat Immunol* 2018, **19**(10):1100-1111.
31. Newman JN, Floyd RV, Fothergill JL: **Invasion and diversity in Pseudomonas aeruginosa urinary tract infections**. *Journal of medical microbiology* 2022, **71**(3).
32. Wang Y, Cela E, Gagnon S *et al*: **Estrogen aggravates inflammation in Pseudomonas aeruginosa pneumonia in cystic fibrosis mice**. *Respiratory Research* 2010, **11**(1):166.
33. Abid S, Xie S, Bose M *et al*: **17β-Estradiol Dysregulates Innate Immune Responses to Pseudomonas aeruginosa Respiratory Infection and Is Modulated by Estrogen Receptor Antagonism**. *Infection and immunity* 2017, **85**(10).
34. Sharma R, Shultz SR, Robinson MJ *et al*: **Infections after a traumatic brain injury: The complex interplay between the immune and neurological systems**. *Brain, behavior, and immunity* 2019, **79**:63-74.
35. Pittet JF, Hu PJ, Honavar J *et al*: **Estrogen Alleviates Sex-Dependent Differences in Lung Bacterial Clearance and Mortality**

Secondary to Bacterial Pneumonia after Traumatic Brain Injury. *Journal of neurotrauma* 2021, **38**(8):989-999.

36. Wroblewski LE, Peek RM, Jr., Wilson KT: **Helicobacter pylori and gastric cancer: factors that modulate disease risk**. *Clinical microbiology reviews* 2010, **23**(4):713-739.
37. Chen C, Gong X, Yang X *et al*: **The roles of estrogen and estrogen receptors in gastrointestinal disease**. *Oncology letters* 2019, **18**(6):5673-5680.
38. Wesołowska M, Pawlik P, Jagodziński PP: **The clinicopathologic significance of estrogen receptors in human gastric carcinoma**. *Biomedicine & Pharmacotherapy* 2016, **83**:314-322.
39. Namikawa K, Tanaka N, Ota Y *et al*: **Genomic features of Helicobacter pylori-naïve diffuse-type gastric cancer**. *The Journal of pathology* 2022, **258**(3):300-311.
40. Kang S, Park M, Cho JY *et al*: **Tumorigenic mechanisms of estrogen and Helicobacter pylori cytotoxin-associated gene A in estrogen receptor α-positive diffuse-type gastric adenocarcinoma**. *Gastric Cancer* 2022, **25**(4):678-696.
41. Boonyanugomol W, Chomvarin C, Sripa B *et al*: **Helicobacter pylori in Thai patients with cholangiocarcinoma and its association with biliary inflammation and proliferation**. *HPB : the official journal of the International Hepato Pancreato Biliary Association* 2012, **14**(3):177-184.
42. Ma F, Yang Y, Wang JD *et al*: **Helicobacter pylori and 17beta-estradiol induce human intrahepatic biliary epithelial cell abnormal proliferation and oxidative DNA damage**. *Hepatobiliary Pancreat Dis Int* 2017, **16**(5):519-527.
43. Wyres KL, Lam MMC, Holt KE: **Population genomics of Klebsiella pneumoniae**. *Nature reviews Microbiology* 2020, **18**(6):344-359.
44. Ali AA, Diebel LN, Liberati DM: **Estrogen modulation of pneumonia? An immunoglobulin A effect**. *J Trauma Acute Care Surg* 2012, **72**(4):908-915.
45. Mahapure K, Singh A: **A Review of Recent Advances in Our Understanding of Neisseria gonorrhoeae**. *Cureus* 2023, **15**(8):e43464.
46. Hakenberg OW, Harke N, Wagenlehner F: **Urethritis in Men and Women**. *European Urology Supplements* 2017, **16**(4):144-148.
47. Kita E, Takahashi S, Yasui K *et al*: **Effect of estrogen (17 beta-estradiol) on the susceptibility of mice to disseminated gonococcal infection**. *Infection and immunity* 1985, **49**(1):238-243.
48. Cantor A, Dana T, Griffin JC *et al*: **Screening for Chlamydial and Gonococcal Infections: Updated Evidence Report and Systematic Review for the US Preventive Services Task Force**. *Jama* 2021, **326**(10):957-966.
49. Esmaeilnejad-Ganji SM, Esmaeilnejad-Ganji SMR: **Osteoarticular manifestations of human brucellosis: A review**. *World journal of orthopedics* 2019, **10**(2):54-62.
50. Gentilini MV, Pesce Viglietti AI, Arriola Benitez PC *et al*: **Inhibition of Osteoblast Function by Brucella abortus is Reversed by Dehydroepiandrosterone and Involves ERK1/2 and Estrogen Receptor**. *Frontiers in immunology* 2018, **9**:88.
51. Scian R, Barrionuevo P, Rodriguez AM *et al*: **Brucella abortus invasion of synoviocytes inhibits apoptosis and induces bone resorption through RANKL expression**. *Infection and immunity* 2013, **81**(6):1940-1951.
52. Gentilini MV, Giambartolomei GH, Delpino MV: **Adrenal Steroids Modulate Fibroblast-Like Synoviocytes Response During B. abortus Infection**. *Front Endocrinol (Lausanne)* 2019, **10**:722.
53. Miller HK, Priestley RA, Kersh GJ: **Q Fever: A troubling disease and a challenging diagnosis**. *Clinical microbiology newsletter* 2021, **43**(13):109-118.
54. Leone M, Honstettre A, Lepidi H *et al*: **Effect of Sex on Coxiella burnetii Infection: Protective Role of 17β-Estradiol**. *The Journal of infectious diseases* 2004, **189**(2):339-345.
55. Burd EM, Hinrichs BH: **Gastrointestinal Infections**: Molecular Pathology in Clinical Practice. 2015 May 19:707-34. doi: 10.1007/978-3-319-19674-9_50.
56. Kim DK, Jeong JH, Lee JM *et al*: **Inverse agonist of estrogen-related receptor γ controls Salmonella typhimurium infection by modulating host iron homeostasis**. *Nature medicine* 2014, **20**(4):419-424.
